# A Radiomics Model Based on Gd-EOB-DTPA-Enhanced MRI for the Prediction of Microvascular Invasion in Solitary Hepatocellular Carcinoma ≤ 5 cm

**DOI:** 10.3389/fonc.2022.831795

**Published:** 2022-05-19

**Authors:** Chengming Qu, Qiang Wang, Changfeng Li, Qiao Xie, Ping Cai, Xiaochu Yan, Ernesto Sparrelid, Leida Zhang, Kuansheng Ma, Torkel B. Brismar

**Affiliations:** ^1^Institute of Hepatobiliary Surgery, Southwest Hospital, Army Medical University, Chongqing, China; ^2^Division of Medical Imaging and Technology, Department of Clinical Science, Intervention and Technology (CLINTEC), Karolinska Institutet, Stockholm, Sweden; ^3^Division of Radiology, Department of Clinical Science, Intervention and Technology (CLINTEC), Karolinska Institutet, Karolinska University Hospital, Stockholm, Sweden; ^4^Department of Radiology, Southwest Hospital, Army Medical University, Chongqing, China; ^5^Department of Pathology, Southwest Hospital, Army Medical University, Chongqing, China; ^6^Division of Surgery, Department of Clinical Science, Intervention and Technology (CLINTEC), Karolinska Institutet, Karolinska University Hospital, Stockholm, Sweden

**Keywords:** radiomics, microvascular invasion, Gd-EOB-DTPA, magnetic resonance imaging, hepatocellular carcinoma, prediction model

## Abstract

**Aim:**

The aim of this study is to establish and validate a radiomics-based model using preoperative Gd-EOB-DTPA-enhanced MRI to predict microvascular invasion (MVI) in patients with hepatocellular carcinoma ≤ 5 cm.

**Methods:**

Clinicopathologic and MRI data of 178 patients with solitary hepatocellular carcinoma (HCC) (≤5 cm) were retrospectively collected from a single medical center between May 2017 and November 2020. Patients were randomly assigned into training and test subsets by a ratio of 7:3. Imaging features were extracted from the segmented tumor volume of interest with 1-cm expansion on arterial phase (AP) and hepatobiliary phase (HBP) images. Different models based on the significant clinical risk factors and/or selected imaging features were established and the predictive performance of the models was evaluated.

**Results:**

Three radiomics models, the AP_model, the HBP_model, and the AP+HBP_model, were constructed for MVI prediction. Among them, the AP+HBP_model outperformed the other two. When it was combined with a clinical model, consisting of tumor size and alpha-fetoprotein (AFP), the combined model (AP+HBP+Clin_model) showed an area under the curve of 0.90 and 0.70 in the training and test subsets, respectively. Its sensitivity and specificity were 0.91 and 0.76 in the training subset and 0.60 and 0.79 in the test subset, respectively. The calibration curve illustrated that the combined model possessed a good agreement between the predicted and the actual probabilities.

**Conclusions:**

The radiomics-based model combining imaging features from the arterial and hepatobiliary phases of Gd-EOB-DTPA-enhanced MRI and clinical risk factors provides an effective and reliable tool for the preoperative prediction of MVI in patients with HCC ≤ 5 cm.

## Introduction

Hepatocellular carcinoma (HCC) is a common gastrointestinal malignant tumor, ranks sixth in incidence rate, and is the fourth leading cause of tumor-related mortality worldwide (1). Liver resection is one of the curative treatments for HCC. Despite recent advances in surgical techniques and perioperative management, HCC still bears a poor prognosis with a 5-year recurrence of 50%–70% after liver resection (2). This also applies to inpatients with early-stage HCC, where a 5-year recurrence rate of as high as approximately 60% has been reported (3).

Microvascular invasion (MVI) has been reported as an independent, well-established risk factor for HCC recurrence and poor overall survival rate (4). The reported incidence rate of MVI ranges between 15% and 57% (5). Patients with MVI experienced an early recurrence compared with those without MVI with a mean time to recurrence of 12 months versus 42 months (4). Therefore, it is of importance to preoperatively identify MVI to optimize the treatment strategy and predict the prognosis. However, the diagnosis of MVI is mainly made postoperatively by a histopathology exam on the excised tumor, which has little or no influence on surgical decision-making. Although radiological features on computed tomography (CT) or magnetic resonance imaging (MRI) such as rim arterial enhancement and non-smooth tumor margin are also applied to predict MVI, a consensus about the efficacy of these features has not been reached (6, 7).

With the development of modern imaging and computing techniques, it might be possible to detect subtle changes in the tumor or its margin. Radiomics, a technique that can extract high-throughput imaging features from routine biomedical images for quantitative analysis, has attracted intensive interest in recent years (8, 9). Because it may provide additional information, radiomics has turned out to be a promising tool for accurate tumor detection, diagnosis, grading, and prognosis prediction in tumors such as rectal cancer and HCC (10–12).

Gd-EOB-DTPA-enhanced MRI is a commonly used modality in diagnosis and characterization of HCC, and is noted for the specific hepatobiliary phase. Taking advantage of radiomics on Gd-EOB-DTPA-enhanced MRI, it has shown encouraging results for MVI prediction at HCC (13, 14). However, previous studies have not limited their data regarding tumor number and tumor size of HCC (11, 15, 16). With improved imaging and the use of screening programs, HCC is increasingly detected at an early stage. The incidence of MVI in HCC ≤ 5 cm, or within Milan criteria, has been reported to be as high as 40% (17). By identifying these patients preoperatively, their management and long-term survival might be improved as alternative treatment options could be considered. Therefore, the aim of this study was to develop and validate a radiomics prediction model based on preoperative Gd-EOB-DTPA-enhanced MRI to predict MVI in patients with a single HCC ≤ 5 cm in diameter.

## Materials and Methods

### Study Design and Patients

This retrospective study was performed at a single tertiary medical center, Southwest Hospital of Army Medical University, Chongqing, China. The research protocol was approved by the Institutional Review Board of the hospital (No. 2017KY50), and written informed consent was waived due to the retrospective nature of the study.

**Figure 1:**
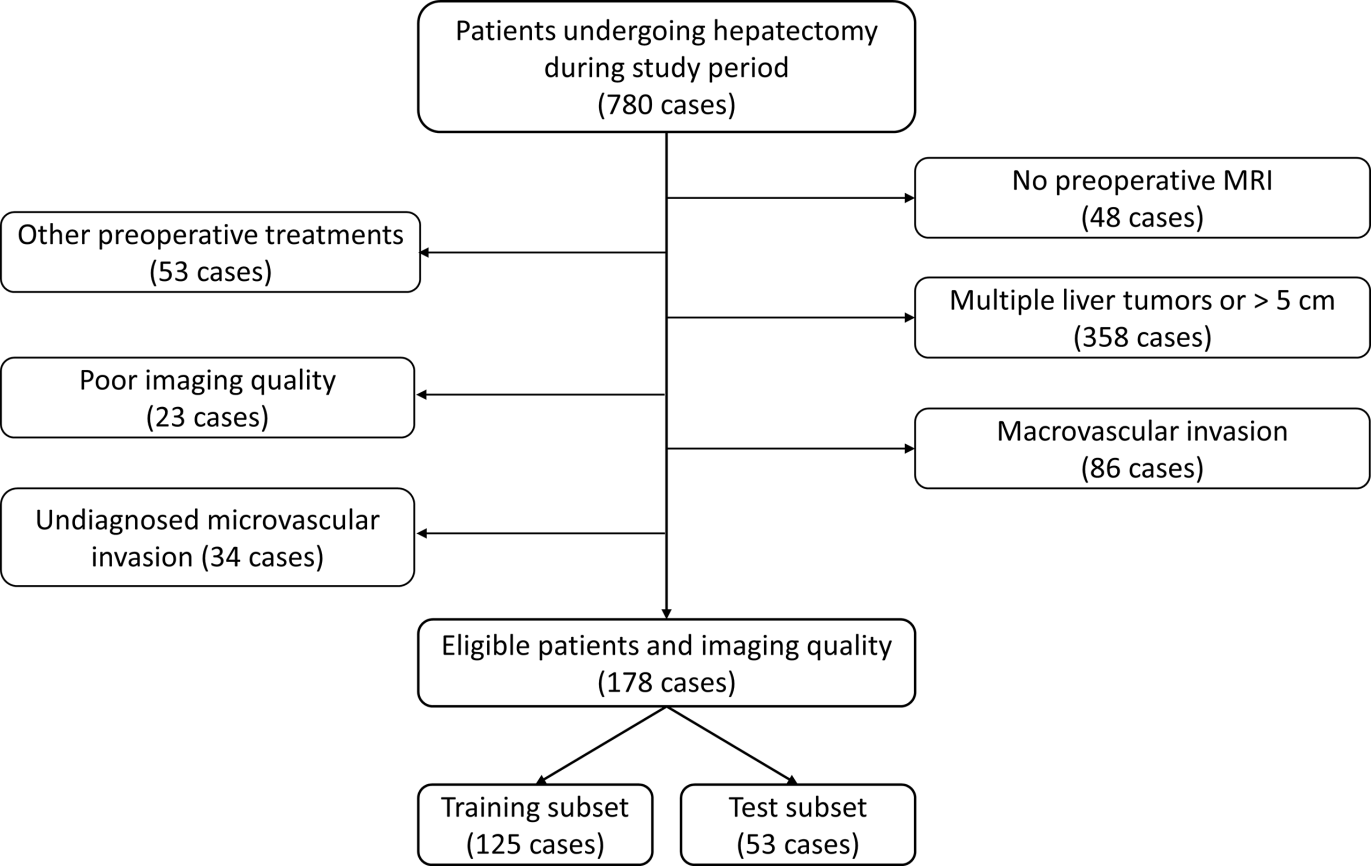
Flowchart of patient selection in this study.

**Figure 2:**
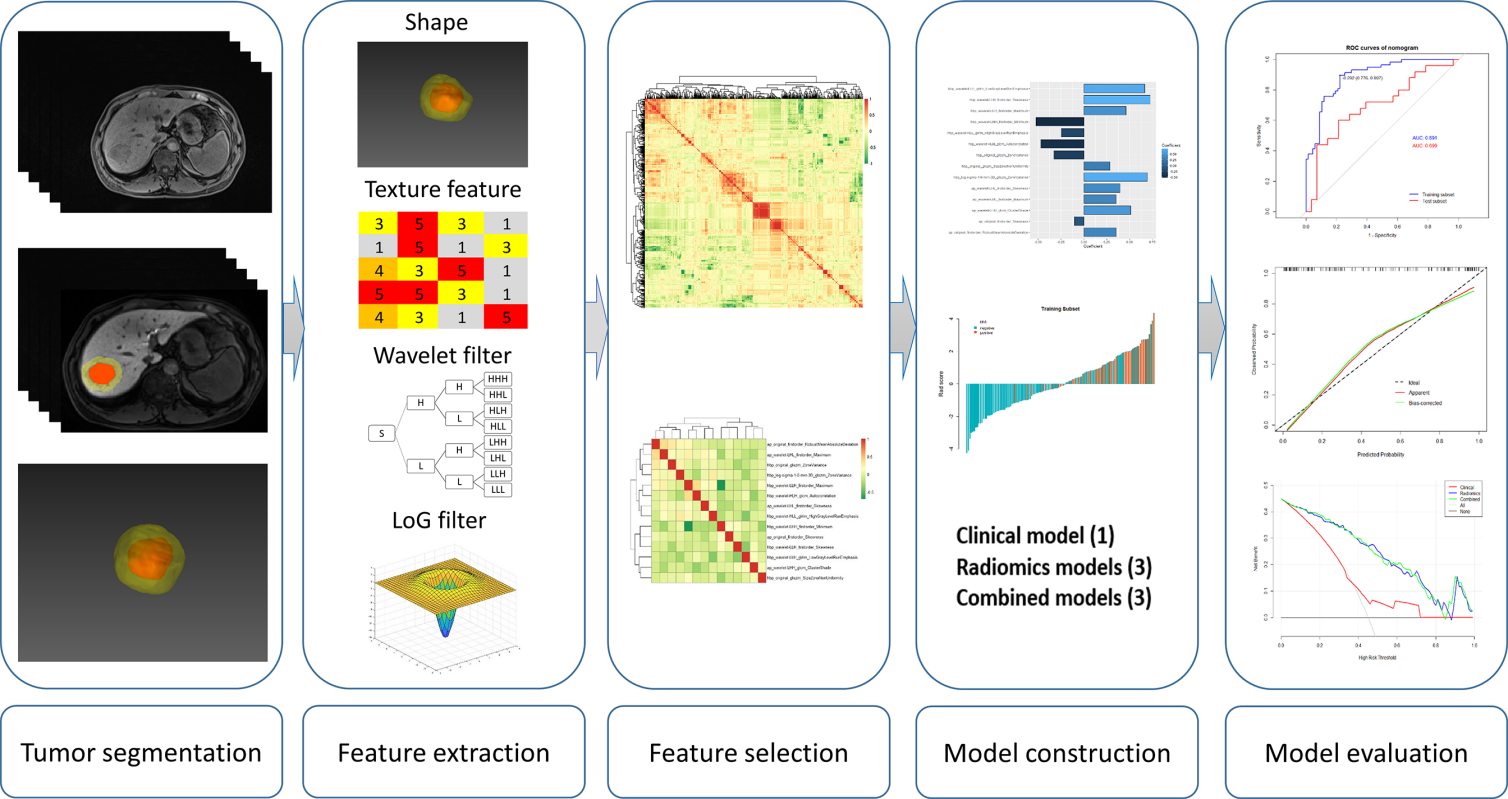
Workflow of key steps in this study.

Through a search in the hospital information system, the records of all patients undergoing liver resection between May 2017 and November 2020 were retrieved. Patients were considered eligible in this study according to the following inclusion criteria: (1) patients undergoing their first liver resection due to HCC, (2) solitary liver tumor with a diameter ≤ 5 cm on MRI with no macroscopic sign of vascular invasion, (3) Gd-EOB-DTPA-enhanced MRI exam within 1 month before liver resection, and (4) available pathology report of MVI status. The exclusion criteria were as follows: (1) previous antitumor treatment, such as radiofrequency ablation and transarterial chemoembolization; (2) intra- or extrahepatic metastasis; and (3) low-quality imaging, not satisfying analysis requirement. A total of 178 consecutive patients were included in the final cohort, and they were randomly split into a training subset and a test subset by a ratio of 7:3. **Figure 1** describes the process of patient selection and **Figure 2** supplies the steps of this study.

### Clinicopathologic Variables and MVI

Demographic information, blood biochemistry, and pathology results were retrieved through the hospital information system. MVI was diagnosed according to the Chinese guidelines for standardized diagnosis of primary liver cancer (18). According to the guidelines, MVI is defined as when cancerous emboli can be observed in the vasculature lined with endothelial cells outside the tumor margin under microscopy (14, 19). In this study, the tumors with this finding were classified as MVI (+), regardless of its number or distance from the tumor. Tumors with no cancerous emboli detected were classified as MVI (−).

### Gd-EOB-DTPA-Enhanced MRI Acquisition

All MRI was performed on the same 3.0-T MRI scanner (Magnetom Trio, Siemens Healthcare) with a 6-channel body coil. The contrast agent Gd-EOB-DTPA (Primovist, Bayer Pharma) was injected through the anterior cubital vein at a dose of 0.1 ml/kg with an injection rate of 1.0 ml/s, followed by an immediate injection of 20 ml of saline at the same rate. After the injection of Gd-EOB-DTPA, arterial phase scanning was triggered by the signal intensity at the lower end of the abdominal aorta, followed by portal phase scanning (60 s), equilibrium phase scanning (180 s), and hepatobiliary phase scanning (15 min) with three-dimensional volume interpolated breath-hold (3D-VIBE) T1WI. The detailed scanning protocol is provided in the **Supplementary Material**.

### Tumor Segmentation and Volume of Interest Dilation

**Figure 3:**
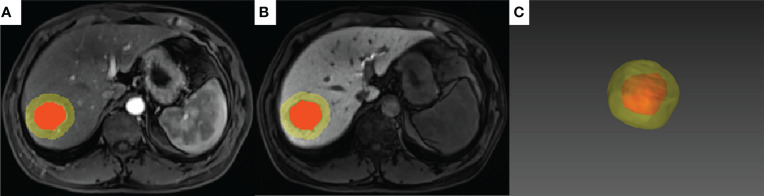
A representative case of tumor segmentation with MVI (+) with 10-mm dilation from the tumor margin. The red area indicates the intratumoral region and the yellow area indicates the peritumoral region on the arterial phase **(A)** and hepatobiliary phase **(B)**. **(C)** 3D effect of the tumor segmentation with 10-mm expansion.

Tumor segmentation on arterial and hepatobiliary phases (hereafter referred to as AP and HBP, respectively) was conducted by two radiologists (QX and PC with 8 and 20 years’ experience in abdominal radiology, respectively) who were blinded to the patients’ clinical information. Tumor delineation was performed manually using the open-source software ITK-SNAP (version 3.8.0, http://www.itksnap.org/). The delineated tumor was further expanded at a radius of 10 mm (20, 21) using a topologic algorithm in Python (version 3.8), and the expansion would cease automatically if it reached the liver edge for the marginal liver tumors. The expanded volume of interest (VOI) was then used for radiomics feature extraction (**Figure 3**).

### Radiomics Feature Extraction

To increase the reliability of the radiomics features, the image voxel size was resampled into 1×1×1 mm^3^ (interpolator: B-spline) and the bin width of the intensity histogram was discretized into 25. After preprocessing the images, the following six categories of imaging features were extracted:

1. Shape, including 2D and 3D (*n* = 14);
2. First-order statistics (*n* = 18);
3. Gray-level co-occurrence matrix (GLCM)-derived texture (*n* = 22);
4. Gray-level run length matrix (GLRLM)-derived texture (*n* = 16);
5. Gray-level size zone matrix (GLSZM)-derived texture (*n* = 16); and
6. Gray-level dependence matrix (GLDM)-derived texture (*n* = 14).

Imaging features of categories (2) to (6) were also extracted from transformed images using the wavelet filter (688 features) and the Laplacian of Gaussian (LoG) filter with a kernel size of 1.0 mm (86 features). Both imaging preprocessing and feature extraction were performed by using the pyradiomics package (version 3.0) (22) in Python (version 3.8).

Imaging features extracted from AP and HBP were labeled with the prefix “ap_” and “hbp_” to each radiomics feature name, respectively. Examples are as follows: “ap_original_firstorder_Skewness” denotes the skewness of first-order features derived from AP images while “hbp_log-sigma-1-0-mm-3D_glszm_ZoneVariance” describes the zone variance in GLSZM features derived from LoG filter transferred HBP images.

To evaluate the reproducibility of the radiomics features and inter-rater agreement, images of 30 randomly chosen patients were contoured by both radiologists independently. The interclass correlation coefficient (ICC) estimates were determined by using the single-rater, absolute-agreement, 2-way random-effects model. The features were classified into “poor-to-moderate” (ICC <0.75) and “good-to-excellent” reliability (ICC ≥ 0.75), and those features with ICC ≥ 0.75 were selected (for the overlapped 30 patients, the measurements of the senior radiologist were selected) for model construction (23).

### Radiomics Model Development and Validation

The AP and HBP imaging features with high reproducibility were adopted for radiomics model establishment. The feature analysis was performed by open-source software that is available at https://github.com/salan668/FAE (24). There was no need for upsampling or downsampling of the data as the percentage of MVI (−) and MVI (+) in the training subset was roughly balanced. The imaging features were first standardized using *z*-score normalization (subtract the mean value of each feature and then divide the difference by its standard deviation), followed by evaluation of the Spearman correlation coefficient of all features. Among each pair of features with a correlation coefficient > 0.90, one was randomly removed. The remaining features were applied for model construction using a recursive feature elimination (RFE) algorithm. RFE iteratively constructs the model using smaller and smaller sets of features and ranks the features according to their importance for the outcome prediction. To avoid potential overfitting, a desired number of features (<20) was applied when establishing the radiomics model using the RFE-logistic regression approach. A 10-fold cross-validation was applied to obtain a stable and robust model. Three radiomics models were constructed using either AP features (AP_model), HBP features (HBP_model), or a combination of both AP and HBP features (AP+HBP_model). Radscore, indicating the relative risk of MVI for each patient, was calculated using each model according to the following formula:


Radscore=intercept+coefficients * features

The predictive performance of the calculated Radscore from each model to predict MVI in training and test subsets was then evaluated.

### Construction and Evaluation of Prediction Models

To establish a clinical model, a univariable logistic regression analysis of the preoperatively clinicopathologic variables including age, gender, etiology of chronic liver disease, cirrhosis status, Child–Pugh grade, tumor size, alpha-fetoprotein (AFP) level, platelet counts, prothrombin time, albumin, bilirubin, aspartate transaminase (AST), and alanine transaminase (ALT) was first applied, with significant risk factors entering the multivariable regression analysis. A clinical model, hereafter denoted Clin_model, was constructed using the significant risk factors observed at the multivariable regression analysis.

The risk factors in the Clin_model and the Radscore were integrated into the construction of three combined models, i.e., AP+Clin_model, HBP+Clin_model, and AP+HBP+Clin_model. The efficacy of these models was validated in the test subset. Calibration curves were plotted to evaluate the predictive performance of the best model in both training and test subsets. Decision curve analysis was performed to evaluate the usefulness of the prediction models.

### Statistical Analysis

Continuous variables were expressed as median with range and tested by Mann–Whitney *U* test to compare the difference between MVI (−) and MVI (+) groups. Categorical variables were presented as number (percentage), and chi-square test or Fisher’s exact test was used to detect the differences between two groups. ICC was calculated by using the package “Pingouin” in Python. The performance of the prediction models was evaluated by receiver operating characteristic curves (ROCs), and the area under ROC (AUC), sensitivity, specificity, positive predictive value, negative predictive value, and accuracy were determined. *p*-values < 0.05 were considered statistically significant. Statistical analyses and randomizations were performed by R software (version 4.0.4, https://www.R-project.org/).

## Results

### Clinicopathologic Characteristics of Patients and Clinical Model

The baseline characteristics of the entire cohort (*n* = 178), the training subset (*n* = 125), and the test subset (*n* = 53) are summarized in **Table 1**. There was no statistically significant difference between the two subsets. The incidence of MVI in the entire cohort was 45.5%.

**Table 1:** Clinicopathologic characteristics of the patients.

Characteristics	Total (*n* = 178)	Training subset (*n* = 125)			Test subset (*n* = 53)			*p*-value^#^
		MVI (−) (*n* = 68)	MVI (+) (*n* = 57)	*p*-value	MVI (−) (*n* = 29)	MVI (+) (*n* = 24)	*p*-value	
Age (years) †	50 (28–78)	51 (30–72)	50 (31–78)	0.576	52 (29–73)	45 (28–72)	0.125	0.449
Gender								
Female	35 (19.7%)	17 (25.0%)	10 (17.5%)	0.429	4 (13.8%)	4 (16.7%)	1.000	0.428
Male	143 (80.3%)	51 (75.0%)	47 (82.5%)		25 (86.2%)	20 (83.3%)		
Etiology								
HBV	169 (94.9%)	66 (97.1%)	54 (94.7%)	0.659	27 (93.1%)	22 (91.7%)	1.000	0.454
None/Others	9 (5.06%)	2 (2.94%)	3 (5.26%)		2 (6.90%)	2 (8.33%)		
Cirrhosis								
Absent	35 (19.7%)	8 (11.8%)	15 (26.3%)	0.063	8 (27.6%)	4 (16.7%)	0.538	0.656
Present	143 (80.3%)	60 (88.2%)	42 (73.7%)		21 (72.4%)	20 (83.3%)		
Child–Pugh Grade								
A	174 (97.8%)	67 (98.5%)	56 (98.2%)	1.000	28 (96.6%)	23 (95.8%)	1.000	0.583
B	4 (2.25%)	1 (1.47%)	1 (1.75%)		1 (3.45%)	1 (4.17%)		
Tumor Size (cm)	3.03 ± 1.09	2.87 ± 1.06	3.29 ± 1.12	0.032*	2.94 ± 1.04	2.98 ± 1.13	0.900	0.564
Tumor Differentiation								
Poor	15 (8.43%)	3 (4.41%)	8 (14.0%)	0.013*	2 (6.90%)	2 (8.33%)	0.162	1.000
Moderate	145 (81.5%)	57 (83.8%)	46 (80.7%)		21 (72.4%)	21 (87.5%)		
Well	14 (7.87%)	8 (11.8%)	1 (1.75%)		5 (17.2%)	0 (0.00%)		
None	4 (2.25%)	0 (0.00%)	2 (3.51%)		1 (3.45%)	1 (4.17%)		
Platelet (×10^9^/L)								
≤125	77 (43.3%)	34 (50.0%)	26 (45.6%)	0.757	9 (31.0%)	8 (33.3%)	1.000	0.073
>125	101 (56.7%)	34 (50.0%)	31 (54.4%)		20 (69.0%)	16 (66.7%)		
Prothrombin time (%)								
≤65	5 (2.81%)	2 (2.94%)	2 (3.51%)	1.000	1 (3.45%)	0 (0.00%)	1.000	1.000
>65	173 (97.2%)	66 (97.1%)	55 (96.5%)		28 (96.6%)	24 (100%)		
Albumin (g/L)								
≤38	31 (17.4%)	10 (14.7%)	11 (19.3%)	0.657	5 (17.2%)	5 (20.8%)	1.000	0.907
>38	147 (82.6%)	58 (85.3%)	46 (80.7%)		24 (82.8%)	19 (79.2%)		
Bilirubin (μmol/L)								
≤21	133 (74.7%)	50 (73.5%)	41 (71.9%)	1.000	23 (79.3%)	19 (79.2%)	1.000	0.474
>21	45 (25.3%)	18 (26.5%)	16 (28.1%)		6 (20.7%)	5 (20.8%)		
ALT (IU/L)								
≤42	119 (66.9%)	45 (66.2%)	36 (63.2%)	0.870	21 (72.4%)	17 (70.8%)	1.000	0.472
>42	59 (33.1%)	23 (33.8%)	21 (36.8%)		8 (27.6%)	7 (29.2%)		
AST (IU/L)								
≤42	135 (75.8%)	52 (76.5%)	40 (70.2%)	0.554	23 (79.3%)	20 (83.3%)	1.000	0.378
>42	43 (24.2%)	16 (23.5%)	17 (29.8%)		6 (20.7%)	4 (16.7%)		
AFP (ng/ml)								
≤400	142 (79.8%)	60 (88.2%)	41 (71.9%)	0.038*	24 (82.8%)	17 (70.8%)	0.482	0.750
>400	36 (20.2%)	8 (11.8%)	16 (28.1%)		5 (17.2%)	7 (29.2%)		

Data are present as number (percentage) except otherwise specified. † Data are expressed as median with range. # Between training and test subsets. AFP, alpha fetoprotein; ALT, alanine transaminase; AST, aspartate transaminase; HBV, hepatitis B virus; MVI, microvascular invasion. *indicates p < 0.05.

After univariable and multivariable regression analyses, two risk factors in the training subset, AFP and tumor size, were selected for clinical model construction (**Table 2**). Although it was significant in both regression analyses, tumor differentiation status was excluded for modeling as it was a postoperative risk factor for MVI prediction. The AUCs of the Clin_model in training and test subsets were 0.64 (95% CI: 0.54–0.74) and 0.55 (95% CI: 0.38–0.71), respectively (**Figure 5A**, **Table 3**).

**Table 2:** Clinical risk factors for MVI presence in patients with hepatocellular carcinoma.

Clinical variable	Univariable analysis		Multivariable analysis	
	OR (95% CI)	*p*-value	OR (95% CI)	*p*-value
Age (years)	0.88 (0.56–1.39)	0.58		
Gender				
Male vs. Female	0.64 (0.27–1.53)	0.32		
Etiology				
HBV vs. None/Others	1.83 (0.30–11.37)	0.52		
Cirrhosis				
Present vs. Absent	2.68 (1.04–6.89)	0.04*	2.39 (0.85–6.74)	0.10
Child–Pugh Grade				
B vs. A	1.20 (0.07–19.57)	0.90		
Tumor Size (cm)	1.79 (1.05–3.06)	0.03*	2.06 (1.15–3.70)	0.02*
Tumor Differentiation				
Moderate vs. Well	0.15 (0.02–1.28)	0.08		
Poor vs. Well	3.30 (0.83–13.17)	0.02*	2.47 (0.54–11.17)	0.03*
Platelet (×10^9^/L)				
>125 vs. ≤125	0.84 (0.41–1.70)	0.63		
Prothrombin time (%)				
>65 vs. ≤65	1.20 (0.16–8.80)	0.86		
Albumin (g/L)				
>38 vs. ≤38	1.39 (0.54–3.55)	0.50		
Bilirubin (μmol/L)				
>21 vs. ≤21	1.08 (0.49–2.39)	0.84		
ALT (IU/L)				
>42 vs. ≤42	1.14 (0.55–2.38)	0.72		
AST (IU/L)				
>42 vs. ≤42	1.38 (0.62–3.07)	0.43		
AFP (ng/ml)				
>400 vs. ≤400	2.93 (1.15–7.47)	0.02*	3.31 (1.20–9.11)	0.02*

AFP, alpha fetoprotein; ALT, alanine transaminase; AST, aspartate transaminase; CI, confidence interval; HBV, hepatitis B virus; MVI, microvascular invasion, OR, odds ratio. *indicates p < 0.05.

**Table 3:** Comparison of the performance of the models in the prediction of MVI presence.

		Clin_model	AP_model	HBP_model	AP+HBP_model	AP+Clin_model	HBP+Clin_model	AP+HBP+Clin_model
Training subset	Cutoff value	−0.06	0.02	−0.56	−0.55	−6.34	−6.16	−6.28
	AUC (95% CI)	0.64 (0.54–0.74)	0.82 (0.75–0.90)	0.87 (0.81–0.93)	0.89 (0.83–0.94)	0.83 (0.77–0.90)	0.87 (0.81–0.93)	0.90 (0.85–0.95)
	Sensitivity	0.51	0.67	0.90	0.91	0.88	0.90	0.91
	Specificity	0.75	0.84	0.68	0.75	0.63	0.68	0.76
	Positive predictive value	0.63	0.78	0.70	0.75	0.67	0.70	0.77
	Negative predictive value	0.65	0.75	0.89	0.91	0.86	0.89	0.91
	Accuracy	0.64	0.76	0.78	0.82	0.74	0.78	0.83
Test subset	AUC (95% CI)	0.55 (0.38–0.71)	0.57 (0.41–0.72)	0.62 (0.47–0.78)	0.66 (0.51–0.81)	0.56 (0.40–0.72)	0.62 (0.47–0.78)	0.70 (0.55–0.84)
	Sensitivity	0.38	0.54	0.71	0.46	0.21	0.63	0.60
	Specificity	0.83	0.62	0.59	0.86	0.97	0.69	0.79
	Positive predictive value	0.64	0.54	0.59	0.73	0.83	0.63	0.71
	Negative predictive value	0.62	0.62	0.71	0.66	0.60	0.69	0.69
	Accuracy	0.62	0.59	0.64	0.68	0.62	0.66	0.70

AUC, area under the receiver operating characteristics curve; CI, confidence interval; MVI, microvascular invasion.

### Feature Selection and Prediction Model Construction

**Figure 4:**
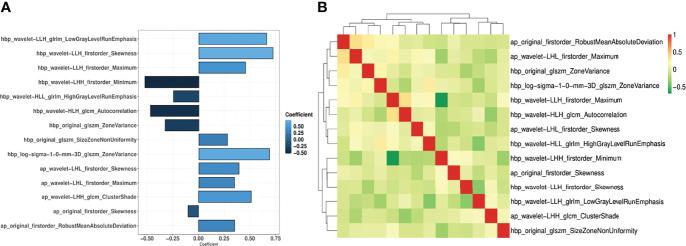
Coefficient of the 14 imaging features **(A)** and the correlation coefficient heatmap **(B)** in the AP+HBP_model.

Out of the 874 imaging features extracted from each Gd-EOB-DTPA-enhanced MRI phase, 560 features (64%) had sufficient reproducibility (ICC ≥ 0.75) for radiomics model construction. After removal of imaging features with high Pearson correlation coefficient, 10 AP features, 12 HBP features, and 14 features from a combination of both AP and HBP features with high ranking selected through the RFE algorithm were used for radiomics model construction (**Figure 4**, **Supplementary File**). The detailed features and their corresponding coefficients for the three radiomics models are described in the **Supplementary File**. The performance of the three radiomics models is illustrated in **Table 3** and the **Supplementary File**.

### Performance Evaluation of the Models

**Figure 5:**
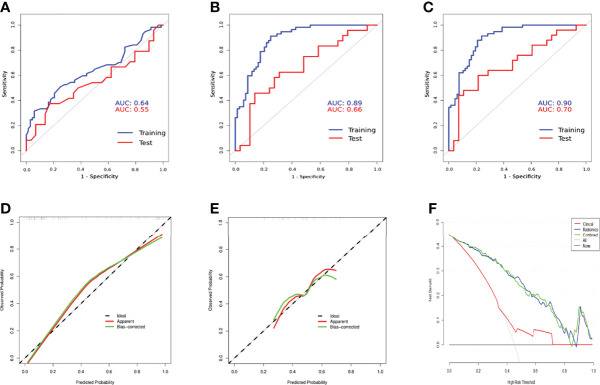
Comparison of receiver operation characteristics curves of the Clin_model **(A)**, AP+HBP_model **(B)**, and AP+HBP+Clin_model **(C)** in training and test subsets. Calibration curves of the AP+HBP+Clin_model for training **(D)** and test **(E)** subsets are shown in the lower left and middle panels. The “apparent” curve (red) represents the prediction model and the bias-corrected curve (green) describes the prediction model calibrated by 1,000 bootstrap samples. The black dashed diagonal line indicates an ideal situation in which the prediction probability is equal to the observed probability. In the lower right panel **(F)**, decision curve analysis for the Clin_model, AP+HBP_model, and AP+HBP+Clin_model is shown. The black line represents the net benefit of assuming that none of the patients have microvascular invasion (MVI), whereas the gray curve represents the net benefit of assuming that all patients have MVI.

Compared with the Clin_model, the overall performance of all three radiomics models was superior, with an AUC above 0.82 in the training subset and more than 0.56 in the test subset. Among the three radiomics models, the AP+HBP_model had the highest AUC, with 0.89 in the training subset and 0.66 in the test subset (**Table 3**). When combined with the clinical variables, the AP+HBP+Clin_model yielded an AUC of 0.90 (95% CI: 0.85–0.95) and 0.70 (95% CI: 0.55–0.84) in the training and test subsets, respectively (**Figures 5B, C**, the formula of the three combined models is provided in the **Supplementary File**). The sensitivity, specificity, positive predictive value, and negative predictive value were 0.91, 0.76, 0.77, and 0.91 in the training subset, and 0.60, 0.79, 0.71, and 0.69 in the test subset, respectively (**Table 3**). The calibration curves illustrated that the predicted probabilities of MVI were in good agreement with the observed probabilities with a C-index of 0.89 and 0.70 in the training and test subsets, respectively (**Figures 5D, E**). In terms of the clinical usefulness evaluation, the decision curve analysis illustrates that the implementation of the AP+HBP+Clin_model to predict MVI status should be beneficial compared with treating none or all of the patients as well as compared with the Clin_model or the AP+HBP_model (**Figure 5F**).

## Discussion

In this study, a radiomics prediction model based on imaging features extracted from preoperative Gd-EOB-DTPA-enhanced MRI to predict MVI in patients with a single HCC ≤ 5 cm in diameter was developed and validated. The best performance was observed when combining imaging features from the arterial and hepatobiliary phases of Gd-EOB-DTPA-enhanced MRI with the two clinical risk factors AFP and tumor size. The predictive value was high, with an AUC reaching 0.90 in the training cohort and 0.70 in the test cohort.

Due to the liver specificity of Gd-EOB-DTPA visualizing the hepatocyte function in the so-called HBP, the differences in texture characteristics between liver tumor and the adjacent tissue are improved. Our results showed that the performance of the HBP_model was better than the AP_model, and hepatobiliary phase features are predominant in the AP+HBP_model (5 vs. 9 features), which gave a clue that the imaging features derived from HBP seem to contain more predictive information. This finding is consistent with two previous studies. In their study, Feng et al. extracted imaging features only from the HBP of Gd-EOB-DTPA-enhanced MRI and constructed a radiomics model showing an AUC of 0.83 in the test cohort, higher than ours (0.83 vs. 0.62) (25). Another research also explored radiomics features on solely HBP images and constructed a prediction model with an AUC of 0.8 (26).

In the AP+HBP_model, the majority of imaging features were derived from wavelet-filtered images, which is in line with previous research (11, 16, 27). This finding implies that the wavelet filter is a powerful tool to obtain decomposition and approximation information of the images. Moreover, most of the imaging features that were included in the model can be categorized into first-order statistics (representing the distribution of voxel intensities), such as maximum, minimum, skewness, and robust mean absolute deviation, indicating that the heterogeneity of the tumor and its surroundings at MRI is associated with MVI presence. This is also in agreement with the abovementioned study by Feng et al., where half of the selected features for modeling belonged to first-order statistics features (25).

As MVI often occurs at the peritumoral area (28, 29), we expanded the tumor margin by 10 mm and extracted the imaging features from intratumoral and peritumoral areas, which we assumed would improve the MVI prediction. The performance of the radiomics models confirmed that assumption. In a similar study, which also constructed models using Gd-EOB-DTPA-enhanced MRI for patients with HCC ≤ 5 cm, the tumor margin was dilated in different diameters, i.e., 5 mm and 10 mm, and also shrunk by 50% (21). The models in that research using features extracted from a combination of the tumor and the 10-mm dilated region yielded an AUC ranging from 0.79 to 0.76 for HBP by two classifiers, random forest and logistic regression, which is a little higher than our model.

Previous studies have attempted to exploit preoperative clinical variables and laboratory tests to predict MVI. Tumor characteristics such as tumor size and tumor number are well-established risk factors for MVI incidence (17, 19). One study with 245 HCC patients undergoing liver transplantation showed that the MVI incidence was 25% in tumors <2 cm, 31% in 2–4 cm tumors, and 50% in tumors >4 cm (30). Another study conducted by Kim et al. demonstrated that the incidence of MVI doubles when there are two or more tumors compared to when there is a solitary HCC (31). Furthermore, the tumor biomarker, AFP, has also been recognized as a reliable predictor for MVI (19, 32, 33). Our Clin_model detected tumor size and AFP as independent risk factors for the prediction model. However, the clinical model using these two risk factors only reached a fair AUC of 0.55 in the test subset. As one of the strategies to improve the performance of a model is to combine variables from different aspects (34), we integrated the clinical risk factors into the AP+HBP_model, improving the AUC to 0.70.

There are some limitations to be acknowledged when interpreting the results of the current study. To begin with, our study was limited by its retrospective nature and sample size. Patient selection bias may thereby have been introduced. Future prospective research should include a larger number of participants to confirm our findings. Moreover, external data from other medical centers are also needed to prove the generalization of our model. Second, although 10-fold cross-validation was adopted during modeling, overfitting might still exist, as seen in the sharp drop of the AUC value in the test subset. Another interpretation for the lower performance in the test subset may be the limited sample size of the test subset, only 53 cases, which makes it sensitive to the performance test. Third, as the current study focused on solitary HCC with a diameter ≤ 5 cm, the generalization of the model needs to be confirmed among HCC patients with no limit for tumor number and size. This should be of special interest when evaluating patients just outside the current transplantation criteria. Furthermore, there are incidence differences among populations due to cirrhosis, viral hepatitis, and nonalcoholic steatohepatitis. This makes it important to validate the model on different cohorts. Fourth, the optimal dilation of the tumor needs to be evaluated as we just dilated the tumor VOIs to 10 mm of the margin as most previously published studies did (20, 21). Future research can be designed to compare different dilations of the tumor diameter when predicting MVI. Fifth, we applied an ICC threshold of 0.75, but the impact of different thresholds on model performance requires further research. Finally, we did not incorporate semantic imaging features, such as the tumor margins or arterial peri-tumoral enhancement, into modeling as we thought those features are more subjective compared with radiomics features. We also did not incorporate images from the portal venous phase due to the same reason as its contrast ratio was inferior to arterial phase. An attempt to build a more objective model using a deep learning approach (without the radiologist’s tumor segmentation) is ongoing in our team.

## Conclusions

Our radiomics-based model combining imaging features from the arterial and hepatobiliary phases of Gd-EOB-DTPA-enhanced MRI and clinical risk factors provides an effective and reliable tool for the preoperative prediction of MVI in patients with HCC ≤ 5 cm.

## Data Availability Statement

The original contributions presented in the study are included in the article/**Supplementary Material**. Further inquiries can be directed to the corresponding authors.

## Ethics Statement

The studies involving human participants were reviewed and approved by the Institutional Review Board of the Southwest Hospital of Army Medical University, Chongqing, China (No.2017KY50). Written informed consent for participation was not required for this study in accordance with the national legislation and the institutional requirements.

## Author Contributions

Writing—original draft preparation: CQ and QW. Writing—review and editing: ES and TBB. Data collection: CQ and CL. Conceptualization: QW, KM, and LZ. Methodology: CQ, QW, QX, and PC. Pathology report interpretation: XY. Formal analysis and investigation: CQ and QW. Funding acquisition: QW and KM. Supervision: TBB, ES, and KM. All authors contributed to the article and approved the submitted version.

## Funding

This work was funded by the National Natural Science Foundation of China (Nos. 82073346 and 81672857), and Famous Teachers section of the Chongqing Talents Program (4246ZP112). QW receives a scholarship from the China Scholarship Council (CSC) (No. 201907930009).

## Conflict of Interest

The authors declare that the research was conducted in the absence of any commercial or financial relationships that could be construed as a potential conflict of interest.

## Publisher’s Note

All claims expressed in this article are solely those of the authors and do not necessarily represent those of their affiliated organizations, or those of the publisher, the editors and the reviewers. Any product that may be evaluated in this article, or claim that may be made by its manufacturer, is not guaranteed or endorsed by the publisher.

## Abbreviations

HCC, Hepatocellular carcinoma; MVI, Microvascular invasion; CT, Computed tomography; MRI, Magnetic resonance imaging; AP, Arterial phase; HBP, Hepatobiliary phase; VOI, Volume of interest; GLCM, Gray-level co-occurrence matrix; GLRLM, Gray-level run length matrix; GLSZM, Gray-level size zone matrix; GLDM, Gray-level dependence matrix; LoG, Laplacian of Gaussian; ICC, Interclass correlation coefficient; RFE, Recursive feature elimination; AFP, Alpha-fetoprotein; AST, Aspartate transaminase; ALT, Alanine transaminase; ROC, Receiver operating characteristic curve; AUC, Area under ROC.
