# Supplementary material for: A Radiomics Model Based on Gd-EOB-DTPA-Enhanced MRI for the Prediction of Microvascular Invasion in Solitary Hepatocellular Carcinoma ≤ 5 cm
Source: Front Oncol. 2022 May 19;12:831795. doi: 10.3389/fonc.2022.831795 (PMC9160991; doi:10.3389/fonc.2022.831795)

**Supplemental Material**

A radiomics model based on Gd-EOB-DTPA enhanced MRI for prediction of microvascular invasion in solitary hepatocellular carcinoma ≤ 5 cm

Chengming Qu, Qiang Wang^*^, Changfeng Li, Qiao Xie, Ping Cai, Xiaochu Yan, Ernesto Sparrelid, Leida Zhang, Kuansheng Ma^*^, Torkel B. Brismar

**Contents**:

1. Gd-EOB-DTPA enhanced MRI scanning parameters

2. The formula of the three radiomics models

3. a. Coefficient of the imaging features (A) and the correlation coefficient heatmap (B) of the AP_model (Supplement Figure 1).

b. Coefficient of the imaging features (C) and the correlation coefficient heatmap (D) of the HBP_model (Supplement Figure 2).

4. The formula of the three combined models

5. Receiver operating characteristic (ROC) curves of the AP_model, HBP_model, AP+Clin_model and AP+HBP_model of training and test subsets (Supplement Figure 3).

**1.** **Gd-EOB-DTPA enhanced MRI scanning parameters**

| Phases | Flip  Angle | Field of  View(mm) | Repetition  time(ms) | Echo  time(ms) | Slice  thickness(mm) |
| --- | --- | --- | --- | --- | --- |
| Arterial phase | 13 | 400×400 | 3.42 | 1.25 | 2.5 |
| Portal phase | 13 | 400×400 | 3.42 | 1.25 | 2.5 |
| Equilibrium phase | 13 | 400×400 | 3.42 | 1.25 | 2.5 |
| Hepatobiliary phase | 30 | 400×400 | 3.42 | 1.25 | 2.5 |

**2. The formula of the three radiomics models**

2.1 Radiomics model (AP_model) using features from arterial phase (AP) images:

AP_model = -0.215+0.122*ap_wavelet-HLL_firstorder_Maximum+

(-0.355)*ap_wavelet-LHL_glcm_Idm+0.680*ap_wavelet-LLL_firstorder_Range+

(-0.638)*ap_original_firstorder_Kurtosis+0.527*ap_wavelet-LHL_firstorder_Skewness+

(-0.288)*ap_wavelet-LHL_gldm_LargeDependenceLowGrayLevelEmphasis+

0.2558381*ap_wavelet-LHL_firstorder_Maximum+

(-0.276)*ap_log-sigma-1-0-mm-3D_glcm_MaximumProbability+

-0.418*ap_original_shape_Flatness+0.651*ap_wavelet-LHH_glcm_ClusterShade

2.2 Radiomics model (HBP_model) using features from hepatobiliary phase (HBP) images:

HBP_model = -0.183+0.857* hbp_wavelet-LLH_firstorder_Skewness+

0.588* hbp_wavelet-LLH_firstorder_Maximum+

0.511*hbp_original_shape_Maximum2DDiameterRow+

0.207* hbp_original_shape_MajorAxisLength+

0.264* hbp_wavelet-HHH_glszm_ZoneVariance+

(-0.452)* hbp_original_glszm_ZoneVariance+

0.800*hbp_wavelet-LLH_glrlm_LowGrayLevelRunEmphasis+

(-0.712)* hbp_wavelet-HLH_glcm_Autocorrelation+

(-0.007)* hbp_wavelet-LHH_firstorder_Range+

0.290*hbp_log-sigma-1-0-mm-3D_glszm_ZoneVariance+

(-0.517)* hbp_wavelet-LHH_firstorder_Minimum+

0.329* hbp_wavelet-LHL_glszm_HighGrayLevelZoneEmphasis

2.3 Radiomics model (AP+HBP_model) using features from arterial and hepatobiliary phases (AP+HBP) images:

AP+HBP_model = -0.208+

0.351*ap_original_firstorder_RobustMeanAbsoluteDeviation+

(-0.102)*ap_original_firstorder_Skewness+

0.395*ap_wavelet-LHL_firstorder_Skewness+

0.351*ap_wavelet-LHL_firstorder_Maximum+

0.513*ap_wavelet-LHH_glcm_ClusterShade+

0.725*hbp_wavelet-LLH_firstorder_Skewness+

0.286*hbp_original_glszm_SizeZoneNonUniformity+

0.460*hbp_wavelet-LLH_firstorder_Maximum+

(-0.247)*hbp_wavelet-HLL_glrlm_HighGrayLevelRunEmphasis+

(-0.327)*hbp_original_glszm_ZoneVariance+

0.664*hbp_wavelet-LLH_glrlm_LowGrayLevelRunEmphasis+

(-0.469)* hbp_wavelet-HLH_glcm_Autocorrelation+

0.691* hbp_log-sigma-1-0-mm-3D_glszm_ZoneVariance+

(-0.523)*hbp_wavelet-LHH_firstorder_Minimum

**3. a. Coefficient of the imaging features (A) and the correlation coefficient heatmap (B) in the AP_model.**

**
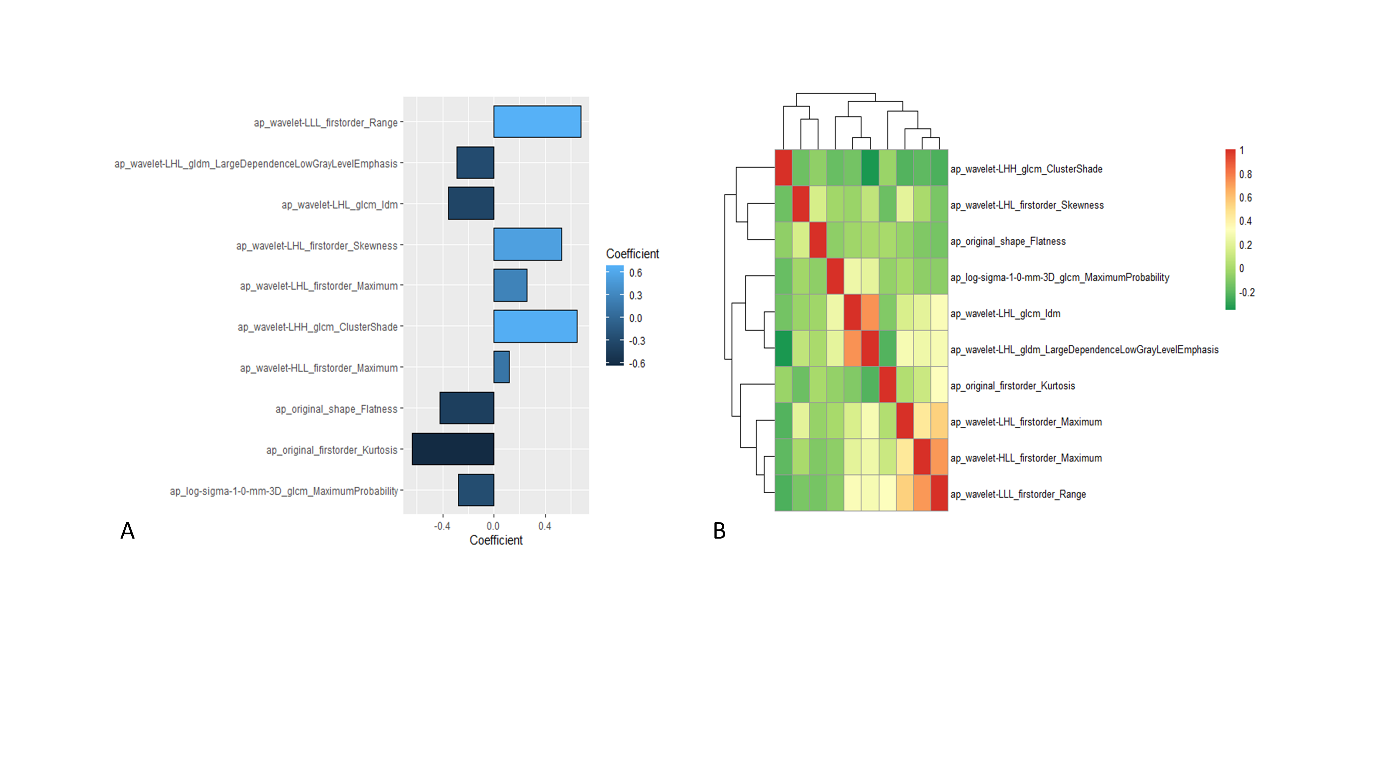
**

**b. Coefficient of the imaging features (A) and the correlation coefficient heatmap (B) in the HBP_model.**


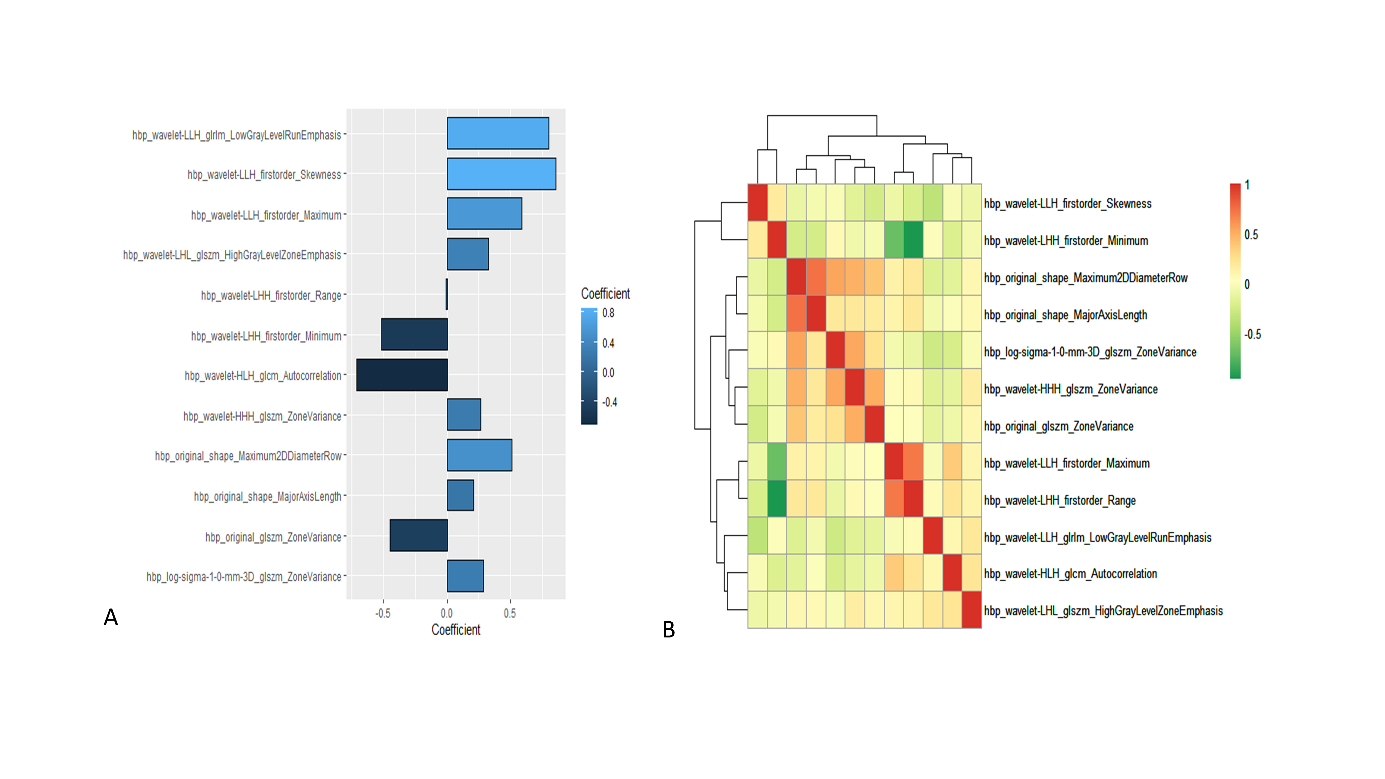


**4. The formula of the three combined models**

The formula for AP+Clin_model is expressed as follows:

Y = -4.0638+1.5358* AFP+0.2624*Tumor size+ 5.8889*Radscore(AP_model)

HBP+Clin_model:

Y = -3.0634+0.4369*AFP+0.0383*Tumor size+5.7123*Radscore(HBP_model)

AP+HBP+Clin_model:

Y = -3.3808+0.0974*Tumor size+0.5931* AFP+ 5.9140* Radscore (AP+HBP_model)

**5. Receiver operating characteristic (ROC) curves of the AP_model, HBP_model, AP+Clin_model and AP+HBP_model in the training and test subsets**

A. AP_model, B. HBP_model, C. AP+Clin_model, D. HBP+Clin_model


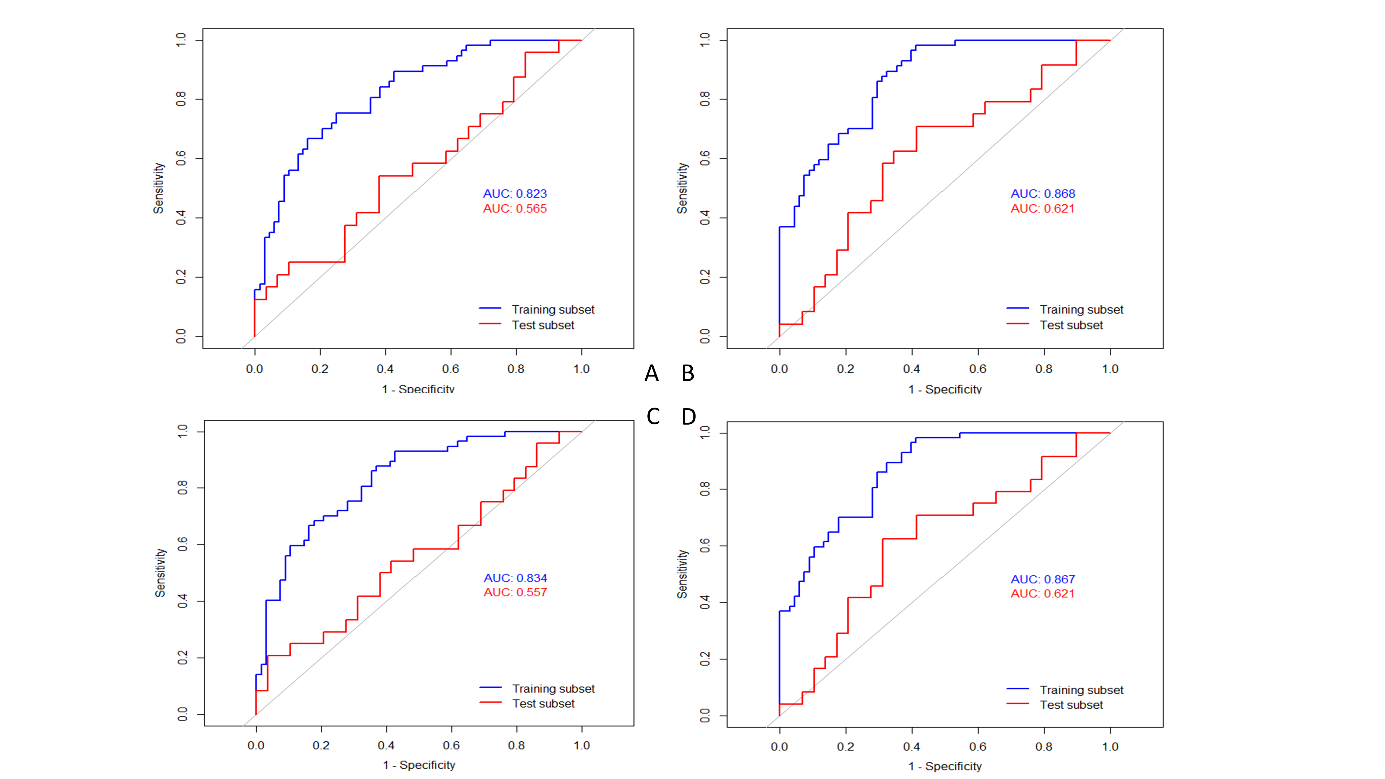

Supplement: Supplementary file 1 [file DataSheet_1.docx]
